# Supplementary material for: Patterns of Intron Gain and Loss in Fungi
Source: PLoS Biol. 2004 Nov 30;2(12):e422. doi: 10.1371/journal.pbio.0020422 (PMC532390; doi:10.1371/journal.pbio.0020422)
Supplement: Table S1 — Also available at http://genes.mit.edu/NielsenEtAl/. (4.3 MB ZIP). [file pbio.0020422.st001.zip › NielsenEtAl/html/653.html]

AN6532.1.NCU00148.1.MG08857.1.FG02599.1


```
 CLUSTAL W (1.82) Multiple Sequence Alignments - Introns Inserted


Sequence 1: NCU00148.1	366 aa
Sequence 2: MG08857.1	387 aa
Sequence 3: FG02599.1	343 aa
Sequence 4: AN6532.1	372 aa
Alignment Length: 410 aa
Number Identitical Residues: 170 aa
Alignment Score (without introns) 8623


MG08857.1 	MDSKATFTNEGAPSEAGSVGNTVISASAKPMKLQLPASCDPPPPQGPTKELVWI0VFGGT
NCU00148.1	-------MEQGTPSITGSET------PSKKGKVALPASCDPPQPGVPRKPLVWI0VFGGT
FG02599.1 	-------MDARTEVSDATEA------SIRPRIAQLPASCDPPQPNAAVKQLVWV0IFGGT
AN6532.1  	------MEFHNSPPRAATALSPSTAVSSREPKLQLPSTTDPPGPQNPPKPLVWL0IFGAT
          	           :    .:  ..  : . :     **:: *** *  . * ***: :**.*

MG08857.1 	GHMGRSLVKCALSHGDSVTTVGKRFESTPEDMVQQH------ERCMGALCDVRIRETVER
NCU00148.1	GQIGRSLVKSCLNHGDFVTIVGRIFESTREQMAAAHLHHPQAENAFGTLCDVRDYASVNR
FG02599.1 	GQMGHTLVNSALAHGDLVTSVGRVFETSLNDMETIH------ENCLGALCDVRARESVNR
AN6532.1  	GHMGRSLVKTALSRDDLVAAVGRTFETSPQAMKKLEDEH---ENCLGLLCDVRARETVQQ
          	*::*::**: .* :.* *: **: **:: : *   .      *..:* *****   :*::

MG08857.1 	VVERTIERFGRVDVIANCSGYGVIGACEDQDEHEIRNQFETNFMGTLHIIQASLPYFRQQ
NCU00148.1	VIDQTLDRFGRIDCIANCSGYGVIGACEDQDEHEIRNQFETNFMGTVHIIQASLPYFREL
FG02599.1 	VIERTLQRFGRIDIVVNCSGYGVIGACEDQDEHDIRNQFETNFMGTLHIIHATLPYFRRQ
AN6532.1  	VIDRTIERFGRIDIIANCAGYGVIGACEDQDEYDIRDQFETNFTGTLNMIQLSLPHFRER
          	*:::*::****:* :.**:*************::**:****** **:::*: :**:**. 

MG08857.1 	AAG-RYLIFSSTAGALGVPGLGPYCSTKYAVEGLIEAMLYEIDSFNLKATLVEPGLVRRD
NCU00148.1	GHGGRYIIFSSTSGALGVPGLGPYCATKYAVEGLIEAMLYEVDAFNIKATLVEPGLVRRD
FG02599.1 	NAG-RYLIFSSTSGALGVPGLGPYCATKYAVEGLIEAMLYETDAFNIKATLIEPGHVRPE
AN6532.1  	RSG-RYLIFSSTSGALGVPGLGPYCASKYAVEGLMESMLYEVDSFNIKTTLVEPGHMRRD
          	  * **:*****:************::*******:*:**** *:**:*:**:*** :* :

MG08857.1 	EPDSQTNPLPT---------------WGHFLIRPASDPYLDPTSPALHAMRMVQWLGDR-
NCU00148.1	EAD--TSMVPR---------------WGHFLIKPASEPYSHATSPALHAKRMVQWLGDK-
FG02599.1 	EPEIQDSTLPTS--------------WGHFLVKPPSETYSNPTAPALHARRIVQWLGDKY
AN6532.1  	DVGDLVSAEPFPSTDSVHNLSSPLPLYGHFLVKPPSEPYNTPTAPAAHAKRMLMWLGDK-
          	:     .  * .::.:  . ::. . :****::*.*:.*  .*:** ** *:: ****: 

MG08857.1 	QPTSAVKCAELVWQLGHCSYPPLRLLLGNYAIESIRDRLRSVTEELEDWKHLNFPAPPDV
NCU00148.1	QPTSAQKCADLVWQLGHCSYPPLRLLLGSYAIESIRDRLRSVTEELEDWKHLHFPVAPEP
FG02599.1 	RPTSAIKCAELVWQLGHCSYPPLRLLLGSYAIESIRDRMRSVTEELEDWKHLNFPAGP--
AN6532.1  	QPASAVKAAHLVWQLGHCSYPPLRLILGTYAVESIRDRLKCIIEEIEDWKYLSFPSMDQP
          	:*:** *.*.***************:**.**:******::.: **:****:* **   . 

MG08857.1 	SDTQENDQEKNERTDTDKDGDKAGGSMEEKMDDAVEDRLDAMDEEMVDFPG
NCU00148.1	GSSGENRGEDGE----NKGSDNVD--RDQDMEMEVETSPTSTKE-------
FG02599.1 	----EDDREAGE---------------EAAMPDANEAAS------------
AN6532.1  	VGSAGQGKDKPT---------------PNVQEQEEEGRT------------
          	 .:  :  :                          *
```
